# Intermittent Fasting Applied in Combination with Rotenone Treatment Exacerbates Dopamine Neurons Degeneration in Mice

**DOI:** 10.3389/fncel.2018.00004

**Published:** 2018-01-17

**Authors:** Giuseppe Tatulli, Nico Mitro, Stefano M. Cannata, Matteo Audano, Donatella Caruso, Giovanna D’Arcangelo, Daniele Lettieri-Barbato, Katia Aquilano

**Affiliations:** ^1^IRCCS San Raffaele La Pisana, Rome, Italy; ^2^Department of Pharmacological and Biomolecular Sciences, University of Milan, Milan, Italy; ^3^Department of Biology, University of Rome Tor Vergata, Rome, Italy; ^4^Department of Medical Systems, University of Rome Tor Vergata, Rome, Italy

**Keywords:** mitochondria, lysophospholipids, excitatory amino acids, Parkinson’s disease

## Abstract

Intermittent fasting (IF) was suggested to be a powerful nutritional strategy to prevent the onset of age-related neurodegenerative diseases associated with compromised brain bioenergetics. Whether the application of IF in combination with a mitochondrial insult could buffer the neurodegenerative process has never been explored yet. Herein, we defined the effects of IF in C57BL/6J mice treated once per 24 h with rotenone (Rot) for 28 days. Rot is a neurotoxin that inhibits the mitochondrial complex I and causes dopamine neurons degeneration, thus reproducing the neurodegenerative process observed in Parkinson’s disease (PD). IF (24 h alternate-day fasting) was applied alone or in concomitance with Rot treatment (Rot/IF). IF and Rot/IF groups showed the same degree of weight loss when compared to control and Rot groups. An accelerating rotarod test revealed that only Rot/IF mice have a decreased ability to sustain the test at the higher speeds. Rot/IF group showed a more marked decrease of dopaminergic neurons and increase in alpha-synuclein (α-syn) accumulation with respect to Rot group in the *substantia nigra* (SN). Through lipidomics and metabolomics analyses, we found that in the SN of Rot/IF mice a significant elevation of excitatory amino acids, inflammatory lysophospholipids and sphingolipids occurred. Collectively, our data suggest that, when applied in combination with neurotoxin exposure, IF does not exert neuroprotective effects but rather exacerbate neuronal death by increasing the levels of excitatory amino acids and inflammatory lipids in association with altered brain membrane composition.

## Introduction

Advanced age is the major risk factor for metabolic and neurodegenerative disorders (Zierer et al., 2015). Mounting evidence from clinical studies and basic research indicate that metabolic dysfunction and brain function decline are causally linked. Type 2 diabetes mellitus appears to be a severe risk factor for developing dementia and neuromotor disease including Parkinson’s disease (PD; Athauda and Foltynie, 2016). Interestingly, insulin resistance in PD patients is associated with a more severe progression of the disease (Athauda and Foltynie, 2016) and insulin resistance has been proposed to be a good predictor of cognitive decline in humans (Ekblad et al., 2017). In experimental models, such phenomenon seems to be caused by increased dopaminergic neuron degeneration. Actually, fat overload in murine models of PD exacerbates neurodegeneration by stimulating iron deposition and specific basal ganglia changes in *substantia nigra* (SN) leading to impaired nigrostriatal dopamine function (Morris et al., 2011; Rotermund et al., 2014).

All these findings lead researchers to study whether reducing calorie intake by caloric restriction or fasting-based dietary strategies could prevent neurodegeneration. The molecular events activated by fasting are conserved from yeast to human and share the ability to protect brain function and metabolic health (Lettieri Barbato et al., 2012, 2015; Lettieri Barbato and Aquilano, 2016). Intermittent fasting (IF) is a generic term used to describe different forms of fasting such as alternate day fasting, periodic fasting, time-controlled fasting. Experimental evidence demonstrates a preventive role of IF in cognitive decline and many neurodegenerative disorders including PD both in rodents and human (Anton and Leeuwenburgh, 2013; Wahl et al., 2016; Mattson et al., 2017).

Omics approaches are an emerging field to evaluate the metabolic changes occurring after treatments or pathophysiological conditions. Lipids are a vast and heterogeneous class of compounds playing a plethora of functions in the brain including myelin formation (Schmitt et al., 2015), signaling (Michel and Bakovic, 2007), inflammation and neuroprotection (Orr et al., 2013) and are strongly related to neurological diseases (Lauwers et al., 2016). Food restriction was effective in preventing phospholipid turnover dysfunction in the brain during aging (Babenko and Shakhova, 2014). Shotgun lipidomics showed that caloric restriction determines important changes in myelin composition in the brain (Kiebish et al., 2012). Specific nutrient restrictions such as methionine restriction have important effects on brain lipid compositions. It has been observed that methionine-restricted mice undergo phospholipid changes and become more resistant to oxidative stress (Jové et al., 2013). This highlights a potential mechanism by which restriction of nutrients may improve brain functionality by phospholipids remodeling. Peculiar increases of brain amino acids, particularly those involved in neurotransmission, define several brain dysfunctions (Sheldon and Robinson, 2007). Notably, fasting strongly impacts on amino acid profile promoting significant reductions in plasma and tissues.

Environmental chemicals represent risk factors for neurodegenerative diseases including PD (Cannon and Greenamyre, 2011). The majority of PD cases are sporadic and chronic exposure to pesticides, such as the complex I inhibitors rotenone (Rot), may play a major role in the pathogenesis (Ascherio and Schwarzschild, 2016). Whether IF may be protective also in this paradigm is currently undefined. Herein, we characterized the effect of IF in modulating brain metabolism and dopaminergic neuron degeneration by using metabolomics approaches. In particular, we treated mice with Rot in combination with IF. Rot is able to induce selective degeneration of dopaminergic neurons and PD-like features in animals (Inden et al., 2011; Tanner et al., 2011). We found that 1 month IF was not sufficient to induce metabolic changes in *SN*; however, IF exacerbated Rot-mediated dopaminergic degeneration that was associated with increased inflammatory phospholipids and excitatory amino acids levels.

## Materials and Methods

### Mice and Treatments

We conducted all mouse experimentations in accordance with accepted standard of humane animal care and with the approval by relevant local (Institutional Animal Care and Use Committee, University of Rome Tor Vergata) and national (Ministry of Welfare, licence no. 331/2016-PR) committees. Experiments were carried out according to institutional safety procedures. Thirty-two C57BL/6J adult (8 weeks-age-old) male mice (purchased from Harlan Laboratories S.r.l., Urbino, Italy) were randomly divided in four groups: control mice (Ctr), IF-treated mice, Rot-treated mice (Rot) and IF/Rot-treated mice (IF/Rot). Rot (30 mg/Kg/day; R8875 Sigma-Aldrich) was dissolved in water supplemented with 4% sodium carboxymethyl cellulose (419273 Sigma-Aldrich) and 3% chloroform and daily dispensed via gavage for 28 days (Inden et al., 2011). The same solution without rotenone was administered to Ctr and IF mice groups. IF treatment was carried out alternating 24 h *ad libitum* food intake with 24 h fasting for 28 days. Rot treatment was started at the first day of fasting and, at the end of treatment, IF and IF/Rot groups were in the fasting state. The Ctr and Rot groups were starved for 2 h, and blood collected to perform bioclinical analyses by colorimetric methods. In particular, glucose, cholesterol, triglycerides, creatinine, creatine phospho-kinase (CPK), alanine transaminase (ALT), glutamic oxaloacetic transaminase (GOT) were measured through the automatized KeyLab analyzer (BPCBioSed, Italy) using specific assay kits (BPCBioSed). Sera Insulin-like growth factor 1 (IGF-1) levels were detected by using Mouse IGF-1 Elisa Kit (Abcam) according to the manufacturer’s instructions. Before sacrifice all mice groups were weighted. After cervical dislocation brain, liver and heart were explanted and weighted. Brains were immediately processed or stored at −80°C.

### Rotarod

Rotarod analyses were performed to test mice latency to fall in order to evaluate the effects on motor coordination. Rotarod analyses were performed both at the baseline prior the first rotenone administration (not included in the results) and 2 days before the end of treatment (at the fasting day and prior rotenone administration). Mice were placed on the rotarod (for mice, Cat N° 47600, Ugo Basile s.r.l., Italy) and sequentially tested at different speeds from 7 rpm to 25 rpm. When mice felt of the rotarod, they were placed back on it for the remaining time. On testing days, each mouse performed one pre-trial (not included in the results) to enable the mice to habituate to the rotarod. The trial was carried out after 2 h of rest.

### Immunohistochemistry

Intact brains were removed and immerged in Carnoy’s fixing solution (60% ethanol, 30% chloroform and 10% glacial acetic acid) overnight at 4°C. Brains were dehydrated with three washes in EtOH absolute, cleared with three washes of Histoclear and embedded in paraffin. Dopaminergic region was identified starting at approximately Bregma −4.30 mm to Bregma −2.63 mm according to Allen Mouse Brain Atlas. This area was cut in 25 μm cross sections with a Leitx rotary microtome. Seventy sections were placed on 13 slides in order to have complete posterior-anterior series and consecutive slides with adjacent sections. Slides were stained with tyrosine-hydroxylase (TH) polyclonal antibody (diluted 1:100, sc-14007 Santa-Cruz Biotechnologies) or alpha-synuclein (α-syn) monoclonal antibody (diluted 1:100, ab138501 Abcam). HRP-conjugated secondary antibodies (1:300 Bio-Rad) and aminoethylcarbazone (AEC Kit, Sigma-Aldrich) as HRP chromogenic substrate were then used.

TH-positive neurons and TH-positive areas were obtained by using ImageJ (Schneider et al., 2012); the sum of each area was multiplied by slides thickness (25 μm) to obtain the volume of SN. α-syn immunostaining was optically quantified for each slide by using ImageJ software according to Mulcahy et al. (2012). Briefly, mean gray values of stained and unstained (background) regions were calculated and converted to optical densities by applying the conversion formula in ImageJ (optical density = log_10_(255/mean gray value). Final optical densities were calculated as the difference in staining between stained and unstained regions. To ensure accuracy of the data, immunohistochemical procedures were simultaneously performed on each sample and all images captured by using Axiolab A1 Zeiss microscope connected to a digital camera (Axiocam ICC5) in the same conditions of bright field illumination and time of exposure.

### Metabolomics

SN were resuspended and lysed in MeOH/ACN 1:1 and spun at 20,000 g for 5 min at 4°C. Supernatant was saved for subsequent analysis. Amino acids quantification was performed by previous derivatization. Briefly, 50 μl of 5% phenyl isothiocyanate (PITC) in 31.5% EtOH and 31.5% pyridine in water were added to 10 μl of each sample. Mixtures were then incubated with PITC solution for 20 min at RT, dried under N_2_ flow and suspended in 100 μl of 5 mM ammonium acetate in MeOH/H_2_O 1:1.

Metabolomic data were performed on an API-4000 triple quadrupole mass spectrometer (AB SCIEX) coupled with a HPLC system (Agilent) and CTC-PAL-HTS auto sampler (PAL System). The identity of all metabolites was confirmed using pure standards. Quantification of different amino acids was performed by using a C18 column (Biocrates). Methanolic samples were analyzed by a 10-min run in positive ion mode with a 20 multiple reaction monitoring (MRM) transition. The mobile phases for positive ion mode analysis (amino acids) were phase A: 0.2% formic acid in water and phase B: 0.2% formic acid in acetonitrile. The gradient was T0 100% A, T5 5 min 5% A, T7 min 100% A with a flow rate of 500 μl/min. MultiQuant™ software (version 3.0.2) was used for data analysis and peak review of chromatograms. Quantitative evaluation of all metabolites was performed based on calibration curves with pure standards, and then data were normalized on protein content.

### Phospholipid Profile by Flow Injection Analysis-Tandem Mass Spectrometry (FIA-MS/MS)

Quantification of total fatty acids was performed as previously described (Cermenati et al., 2017). Briefly, different phospholipid family quantification was performed by flow injection analysis-tandem mass spectrometry (FIA-MS/MS) method. The identity of the different phospholipid families was confirmed using pure standards, namely one for each family. Methanolic extracts were analyzed by a 3-min run in both positive and negative ion mode with a 268 MRM transition in positive mode and 88 MRM transition in negative mode. An ESI source connected with an API 4000 triple quadrupole instrument (AB Sciex, Framingham, MA, USA) was used. The mobile phase was 0.1% formic acid in MeOH for FIA positive analysis and 5 mM ammonium acetate pH 7 in MeOH for FIA negative. MultiQuant™ software version 3.0.2 was used for data analysis and peak review of chromatograms. Semiquantitative evaluation of PL families was performed based on external standards.

### Statistical Analysis

Statistical significance of the differences between group sample mean values were determined by analysis of variance (ANOVA) followed by the Student-Newman-Keuls test for pairwise comparison of means. All statistical analyses were carried out by using GraphPad Prism 5 Software (GraphPad Software). The results are presented as mean ± SD. Differences were considered to be significant at *p* < 0.05.

## Results

**Figure 1:**
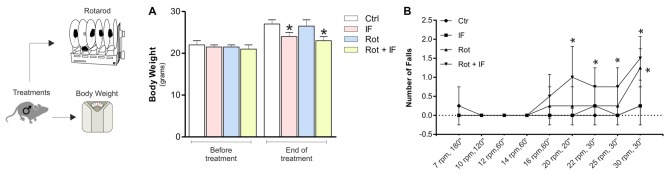
Intermittent fasting (IF) causes motor dysfunction in rotenone-treated mice. **(A)** Body weight was recorded immediately before and at the end of treatment. Data are expressed as means ± SD (*n* = 4; *F*_(7,8)_ = 5.60; *p < 0.05 vs. Ctr). **(B)** A rotarod test was performed at different times and speeds to evaluate motor function (*n* = 4, *F*_(3,12)_ = 4.01; *p < 0.05 vs. Ctr).

To reproduce brain mitochondrial dysfunction, we treated mice once per 24 h with Rot for 28 days according to Inden et al. (2011). Treatment with the pesticide Rot in rodents inhibits mitochondrial complex I and induces striatal degeneration thus reproducing features of age-related neurological disorders such as PD and parkinsonisms (Costa et al., 2008). Furthermore, the Rot-treated mouse has been suggested to provide an interesting animal model for preclinical examinations of neuroprotective strategies (Inden et al., 2011). To evaluate the effects of IF, we compared Rot-treated (Rot) mice with a group of Rot-treated mice subjected to IF (Rot/IF). In line with other studies, Rot did not cause changes in body weight (Inden et al., 2011; Liu et al., 2015), whereas IF caused about 10% decrease of body weight that was comparable to that observed in Rot/IF-treated mice (Figure 1A). These findings are in line with previous works revealing the slimming effect of IF (Lettieri-Barbato et al., 2016). No alterations in brain weights and other organs, such as liver and heart, were instead observed (data not shown). Bioclinical analyses reported in Table 1 revealed that all groups did not manifest metabolic alterations as assessed by analyzing fasting glycaemia, blood cholesterol and triglycerides. Also, the analysis of blood creatinine, CPK, ALT, GOT indicated that kidney, skeletal muscle and liver damage were not induced. The analysis of serum IGF-1 levels showed that they were decreased both in IF and Rot/IF groups with respect to Ctr and Rot groups (Table 1). This result confirmed the effectiveness of IF, as lowered IGF-1 levels are found upon different dietary restriction regimens in rodents and human (Mitchell et al., 2010; Lettieri-Barbato et al., 2016).

**Table 1:** Clinical biochemistry parameters and serum insulin-like growth factor 1 (IGF-1) levels.

	Ctr	IF	Rot	Rot/IF
Glucose (mg/dl) 60–120	82.00 ± 13.49	78.83 ± 16.67	80.83 ± 19.34	87.0 ± 10.95
Creatinine (mg/dl) 0.50–1.20	0.82 ± 0.08	0.86 ± 0.22	0.78 ± 0.05	0.92 ± 0.18
Cholesterol (mg/dl) 50–120	71.00 ± 17.37	84.0 ± 20.71	77.66 ± 7.71	82.6 ± 12.52
Triglycerides (mg/dl) 80–200	85.33 ± 6.05	83.83 ± 2.14	88.17 ± 6.94	85.2 ± 3.35
AST (U/L) 20–250	54.17 ± 13.65	80.00 ± 40.48	49.00 ± 8.15	40.8 ± 16.51
Alanine transaminase (ALT) (U/L) 20–100	26.38 ± 4.48	24.48 ± 1.70	26.60 ± 4.06	26.54 ± 5.65
Creatine phospho-kinase (CPK) (U/L) 100–300	219.50 ± 61.08	162.00 ± 67.05	254.17 ± 67.62	234.4 ± 60.57
IGF-1 (pg/ml)	1005 ± 40	866 ± 30*	1100 ± 118	901 ± 32^#^

Mice were treated daily with rotenone (Rot) concomitant to normal feeding with control diet or intermittent fasting (IF, 24 h alternate-day fasting) for 28 days. Prior sacrifice, mice were starved for 2 h and blood was collected for clinical biochemistry analyses and for assessing IGF-1 levels (*n* = 6 each group, *p = 0.013, ^#^*p* = 0.034 vs. Ctr).

To assess the possible acquisition of motor dysfunction, we carried out an accelerating rotarod test. As reported in Figure 1B, IF did not lead to alteration in the motor function, while a significant decrease in the mouse ability to sustain the test was observed in Rot and Rot/IF group. However, the Rot/IF mice started to manifest the motor dysfunction at minor speeds (20 rpm, 30 s) with respect to Rot group (30 rpm, 30 s) as showed in Figure 1B.

**Figure 2:**
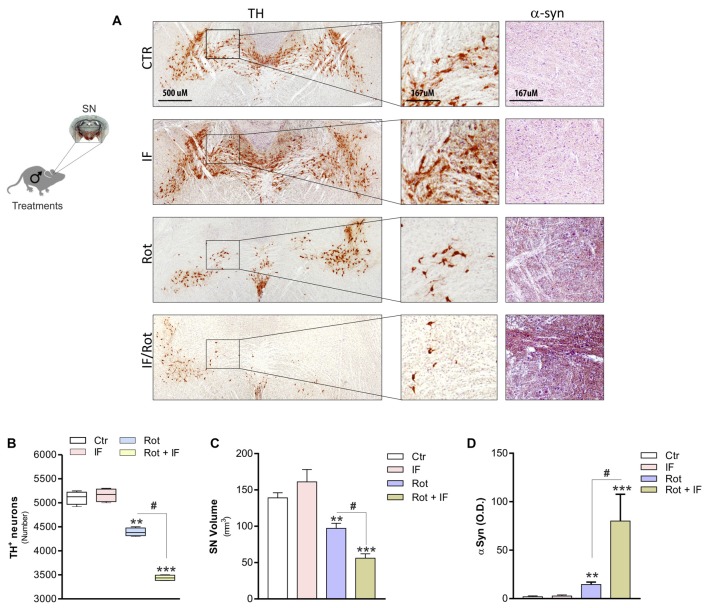
IF enhances neurodegeneration caused by rotenone treatment.** (A)** Brains were fixed and used for immunohistochemistry analysis of dopaminergic neurons and α-synuclein (α-syn) by staining through tyrosine hydroxylase (TH) and synuclein antibody, respectively. Images reported are from one mice of each group and representative of four giving similar results. **(B)** Total TH positive (TH^+^) neurons in the slices including *substantia nigra* (SN) were counted for each group by using ImageJ software (*F*_(3,12)_ = 220.20; **p < 0.01, ***p < 0.001 vs. Ctr; ^#^*p* < 0.01 vs. Rot). **(C)*** SN* volume was calculated for each group by using ImageJ software (*F*_(3,4)_ = 20.44; **p < 0.01, ***p < 0.001 vs. Ctr; ^#^*p* < 0.01 vs. Rot). **(D)** α-syn immunoreactivity was quantified for each group by using ImageJ software and expressed as optical density (*F*_(3,28)_ = 7.16; **p < 0.01, ***p < 0.001 vs. Ctr; ^#^*p* < 0.01 vs. Rot). Data are expressed as means ± SD (*n* = 4 each group).

To validate the occurrence of anatomical lesions in dopaminergic area we next performed an immunohistochemistry analysis of TH-positive neurons. Rot treatment induced significant changes in the SN. Actually, we observed a decrease in the number of TH-positive neurons (Figures 2A,B) accompanied by a reduction in overall SN volume (Figure 2C) with respect to controls. Notably, this tissue damage was higher in Rot/IF with respect to Rot-treated mice (Figures 2A–C). Differently, IF did not induce changes in the number of TH-positive neurons and SN volume (Figures 2A–C). We also performed an immunohistochemistry determination of α-syn. According to the data obtained analyzing TH-positive neurons, we found that α-syn accumulation was well detectable in Rot-treated group and its immunoreactive levels were further increased when Rot was used in combination with IF (Figures 2A,D).

**Figure 3:**
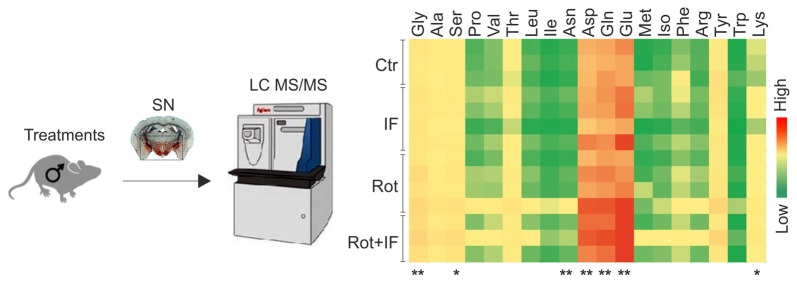
Exacerbation of rotenone-mediated dopaminergic degeneration is associated with increased excitatory amino acids levels in mice subjected to IF. Brain region containing *SN* were analyzed by LC-MS/MS. Heat map reports amino acids levels for each group. Gly (*F*_(3,8)_ = 6.77, **p < 0.01 Rot + IF vs. Ctr), Ser (*F*_(3,8)_ = 5.33, *p < 0.05 Rot + IF vs. Ctr), Asn (*F*_(3,8)_ = 4.59, **p < 0.01 Rot + IF vs. Ctr), Asp (*F*_(3,8)_ = 7.37, **p < 0.01 Rot + IF vs. Ctr), Gln (*F*_(3,8)_ = 10.31, **p < 0.01 Rot + IF vs. Ctr), Glu (*F*_(3,8)_ = 18.88, **p < 0.01 Rot + IF vs. Ctr), Lys (*F*_(3,8)_ = 5.81, *p < 0.05 Rot + IF vs. Ctr). Data are expressed as means ± SD (*n* = 3 for Rot + IF group; *n* = 4 for the other groups).

Amino acids are important players in neurotransmission and take center place in excitatory pathways (Shaw, 1992). Altered levels of excitatory amino acids characterize several neurodegenerative disorders including PD (Salińska et al., 2005; Blandini, 2010). Thus, we measured the levels of all the amino acids in the SN-enriched region of the brain. Among the detectable amino acids, we found increased levels of glutamate, glutamine, aspartate, glycine only in Rot/IF group, whereas no amino acids variations were observed in other groups (Figure 3).

**Figure 4:**
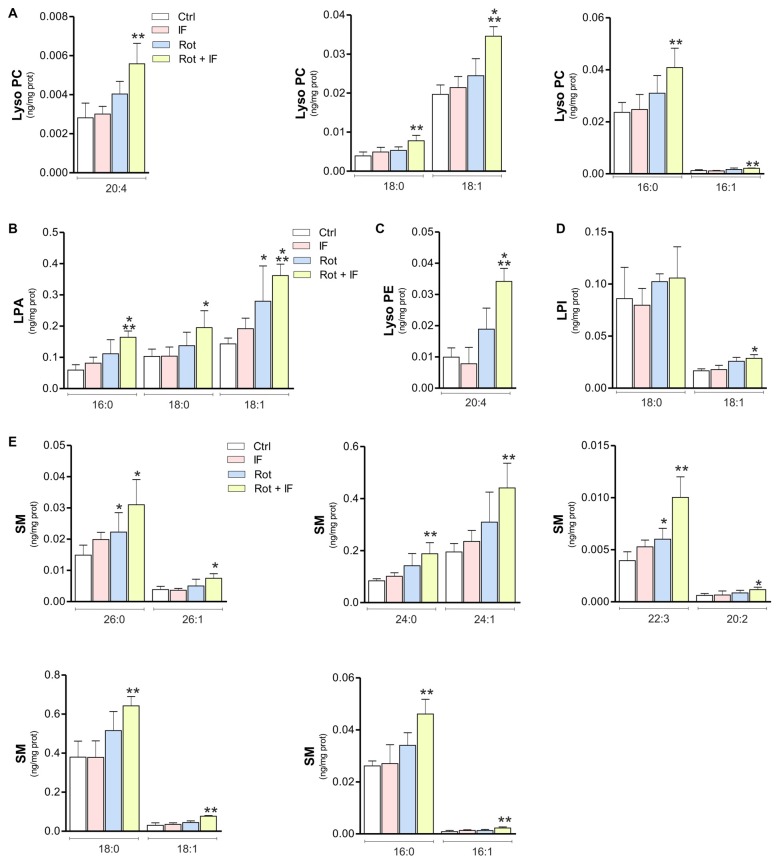
Levels of inflammatory lysophospholipids and sphingomyelin (SM) are increased in mice treated with rotenone and subjected to IF. Brain regions containing *SN* were analyzed by LC-MS/MS. Levels of LPC coline species 20:4 (*F*_(3,11)_ = 10.54; **p < 0.01 vs. Ctr), LPC 18:0 (*F*_(3,11)_ = 11.52; **p < 0.01 vs. Ctr), LPC 18:1 (*F*_(3,12)_ = 103.61; ***p < 0.001 vs. Ctr), LPC 16:0 (*F*_(3,12)_ = 13.72; **p < 0.01 vs. Ctr) and LPC 16:1 (*F*_(4,12)_ = 23.72; **p < 0.01 vs. Ctr) **(A)**; lysophosphatidic acid (LPA) species LPA 16:0 (*F*_(3,11)_ = 8.71; ***p < 0.001 vs. Ctr), LPA 18:0 (*F*_(3,11)_ = 4.38; *p < 0.05 vs. Ctr) and LPA 18:1 (*F*_(3,11)_ = 7.88; *p < 0.05, ***p < 0.001 vs. Ctr) **(B)**; lysophosphatidyl ethanolamine (LPE) specie 20:4 (*F*_(3,7)_ = 12.88; ***p < 0.001 vs. Ctr) **(C)**; lysophosphatidyl inositol (LPI) species LPI 18:0 and LPI 18: 1 (*F*_(3,11)_ = 10.63; *p < 0.05 vs. Ctr) **(D)**; SM species SM 26:0 (*F*_(3,11)_ = 5.78; *p < 0.05 vs. Ctr), SM 26:1 (*F*_(3,11)_ = 4.97; *p < 0.05 vs. Ctr), SM 24:0 (*F*_(3,11)_ = 7.39; **p < 0.01 vs. Ctr), SM 24:1 (*F*_(3,11)_ = 8.49; **p < 0.001 vs. Ctr), SM 22:3 (*F*_(3,11)_ = 17.14; *p < 0.05, **p < 0.01 vs. Ctr), SM 20:2 (*F*_(3,11)_ = 2.78; *p < 0.05 vs. Ctr), SM 18:0 (*F*_(3,11)_ = 8.01; **p < 0.01 vs. Ctr), SM 18:1 (*F*_(3,11)_ = 17.56; **p < 0.01 vs. Ctr), SM 16:0 (*F*_(3,11)_ = 10.25; **p < 0.01 vs. Ctr) and SM 16:1 (*F*_(3,11)_ = 8.49; **p < 0.01 vs. Ctr) **(E)**. Data expressed as means ± SD (*n* = 3 for Rot + IF group; *n* = 4 for the other groups).

Brain phospholipid composition could be significantly altered as consequence of neuronal loss and inflammatory processes (Farooqui et al., 2004). Thus, we analyzed phospholipid composition of SN-enriched brain regions. The obtained lipidomic profile identified 218 phospholipids. Among these, 10 were up-regulated in IF, about 74 in Rot, 152 in Rot/IF group. Sixty-nine phospholipids were in common between Rot and Rot/IF group and 60 were up-regulated exclusively in the Rot/IF group. Regarding Rot/IF group, the up-regulated phospholipids belonged to all the species but not sulfatide (data not shown) and the most affected classes (with 100% of the detected species increased) were sphingomyelin (SM) and the lysophospholipids i.e., lysophosphatidic acid (LPA), lysophosphatidylcholine (LPC) and lysophosphatidylethanolamine (LPE; Supplementary Figure S1 and Figure 4).

## Discussion

In this work, we reproduced PD-like features through Rot treatment in mice and demonstrated that the concomitant administration of IF imposes significant metabolic alterations in SN-enriched regions that are associated with the aggravation of dopamine neuron degeneration as well as motor dysfunction.

Our results are in contrast with many others showing that dietary restriction exerts a neuroprotective function. In these studies, dietary restriction was applied for very long periods prior to a neurotoxic insult allowing the activation of neuronal stress responses that could mitigate the neurodegenerative processes. In particular, IF was found to protect hippocampal or dopaminergic neuron degeneration when applied prior excitotoxic insults or mitochondrial toxin 1-methyl-4-phenyl-1,2,3,6-tetrahydropyridine (MPTP) treatment in rodents (Bruce-Keller et al., 1999; Duan and Mattson, 1999). Pre-treatment with dietary restriction was also found to protect dopaminergic neurons in a murine PD model using lactacystin (Coppens et al., 2017). Attenuated motor deficits were observed in monkeys that were fed with 30% of calorie reduction for 6 months prior to treatment with MPTP (Maswood et al., 2004). The protective effects of dietary restriction have been ascribed to its ability to lower mitochondrial reactive oxygen species production and raise antioxidant levels (Walsh et al., 2014) or to enhance the production of neurotrophic factors (i.e., glial cell line-derived neurotrophic factor, brain-derived neurotrophic factor) with consequent activation of neuroprotective signal transduction pathways in domaminergic neurons (Maswood et al., 2004).

A study from the group of Armentero et al. (2008) showed that dietary restriction was instead not able to prevent the degeneration of dopaminergic neurons in rodents treated with the PD toxin 6-hydroxydopamine. These authors suggested that the lack of protection was due to the too short period of dietary restriction regimen preceding 6-hydroxydopamine treatment that did not allow the development of stress resistance signaling pathways.

To the best of our knowledge, our work represents the first study investigating the effect of IF concomitant to an environmental insult and demonstrating a deleterious effect of IF. We show that even though IF lowers body weight and IGF-1 plasma levels, IF alone did not produce significant alterations in SN. Intriguingly, when administered in combination with Rot treatment, IF leads to significant increase of excitatory amino acids as well as phospholipids levels in SN and exacerbates dopamine neurons degeneration, arguing that IF was likely unable to activate neuroprotective response against Rot toxicity.

The loss of dopamine due to the degeneration of the neurons in the SN elicits alterations of the neurotransmitter circuitry in the basal ganglia (Calabresi et al., 2007; Obeso et al., 2008) and, as consequence, an imbalance of neurotransmission may develop in the nigrostriatal system (Cobb and Abercrombie, 2003; Bradner et al., 2013). Amino acids play an important role in the metabolism of neurotransmitters. When present at high levels in the brain they can be toxic to neurons, a phenomenon known as “excitotoxicity”. N-methyl-D-aspartate ionotropic glutamate receptor (NMDA) is a ligand-gated ion channel that is crucial for excitatory neurotransmission and its excessive activation plays a key role in mediating some aspects of synaptic dysfunction in several neuronal disorders including PD (Lau and Tymianski, 2010). Increased excitatory neurotransmitters have been found in plasma and brain of PD patients and mice models (Iwasaki et al., 1992; Assous et al., 2014). Glutamate is the predominant excitatory neurotransmitter in the central nervous system (Meldrum, 2000). Glutamine represents a precursor of the excitatory transmitter glutamate and aspartate (Holten and Gundersen, 2008). Glycine exerts excitatory effects in the cortex as well as hippocampal neurons by acting as a NMDA receptor co-agonist increasing the affinity of NMDA receptors for glutamate (Fadda et al., 1988; Barth et al., 2005). With all this in mind, it is conceivable that the exacerbation of Rot-mediated neurodegeneration triggered by IF could at least in part depend on the increase of excitatory amino acids that we observed in SN.

Changes in the lipidome profile could occur in regions undergoing significant neuronal loss and inflammation. Actually, a number of phospholipids are involved in inflammatory cascades, and maintenance of phospholipids homeostasis is fundamental for assuring physiological function of cell membrane of brains cells (Posse de Chaves and Sipione, 2010; Aureli et al., 2015). In addition to the degeneration of dopaminergic system, gliosis is a prominent feature of PD (Członkowska and Kurkowska-Jastrzębska, 2011) that can be observed also in PD animal models (Strömberg et al., 1986). Our data suggest that IF worsens the harmful effects of Rot by favoring lipid membrane disturbances and raising the brain concentration of potentially inflammatory and neurotoxic phospholipids. In support of this assumption, elevated LPA and LPC were found to lead to brain injury (Tigyi et al., 1995; Goldshmit et al., 2010) and are likely involved in the pathogenesis of PD (Lee et al., 2005). Furthermore, it has been reported that elevated LPC levels in the brain are potent attraction signals for phagocytes such as activated microglia, astroglia and macrophages (Zhang et al., 2007). The increased levels of SM could imply a disruption of cell membranes, and in particular myelin composition, and a pro-inflammatory state. Interestingly, aberrant accumulation of sphingolipids in the prefontal cortex may also contribute to dysregulation of synaptic vesicle fusion and trafficking of NMDA receptors (Haughey et al., 2010), thus explaining the detected imbalance of excitatory amino acid profile.

The exact molecular mechanisms by which, in our model, IF sensitizes dopaminergic neurons to the toxic effects of Rot remain to be more deeply elucidated yet and are currently under investigation in our laboratory. A hypothetical explanation could be that upon fasting neurons have scarce levels of nutrients and the concomitant inhibition of complex I by Rot triggers a harmful energetic drop. It is now widely accepted that excessive NMDA receptors stimulation aggravates PD via the enhancement of mitochondrial damage (Greenamyre et al., 1999). An increase of inflammatory lipids could also contribute to damage mitochondria (Currais, 2015) and further create an energetic drop that sensitizes neurons to Rot upon IF. Based on this evidence, we can speculate that an increased death of dopaminergic neurons could be operative in Rot/IF group and at least in part due to an energetic crisis.

In conclusion, even though our results remain correlative, as we have not tested the level of astrocytes and microglia markers as well as dopaminergic neuron death, our work indicates that IF should not to be always considered of safe application but be followed with caution possibly only when external insults are limited; indeed, it may produce important side-effects, such as elevation of inflammatory lipids and excitatory amino acids levels that could exacerbate dopaminergic neurons degeneration and accelerate brain aging.

## Author Contributions

KA and DL-B designed the research and wrote the manuscript. GT, MA, GD and SMC performed the research. NM and DC analyzed the data.

## Conflict of Interest Statement

The authors declare that the research was conducted in the absence of any commercial or financial relationships that could be construed as a potential conflict of interest. The reviewer WV and handling Editor declared their shared affiliation.

## Abbreviations

α-syn: alpha-synuclein
ALT: alanine transaminase
CPK: creatine phospho-kinase
GOT: glutamic oxaloacetic transaminase
IGF-1: Insulin-like growth factor 1
IF: intermittent fasting
LPA: lysophosphatidic acid
LPC: lysophosphatidylcholine
LPE: lysophosphatidylethanolamine
MPTP: 1-methyl-4-phenyl-1,2,3,6-tetrahydropyridine
NMDA: N-methyl-D-aspartate ionotropic glutamate receptor
PD: Parkinson’s disease
Rot: rotenone
SN: substantia nigra
TH: tyrosine hydroxylase.
