# Supplementary material for: Intermittent Fasting Applied in Combination with Rotenone Treatment Exacerbates Dopamine Neurons Degeneration in Mice
Source: Front Cell Neurosci. 2018 Jan 17;12:4. doi: 10.3389/fncel.2018.00004 (PMC5776087; doi:10.3389/fncel.2018.00004)
Supplement: Supplementary file 1 [file Image_1.pdf]

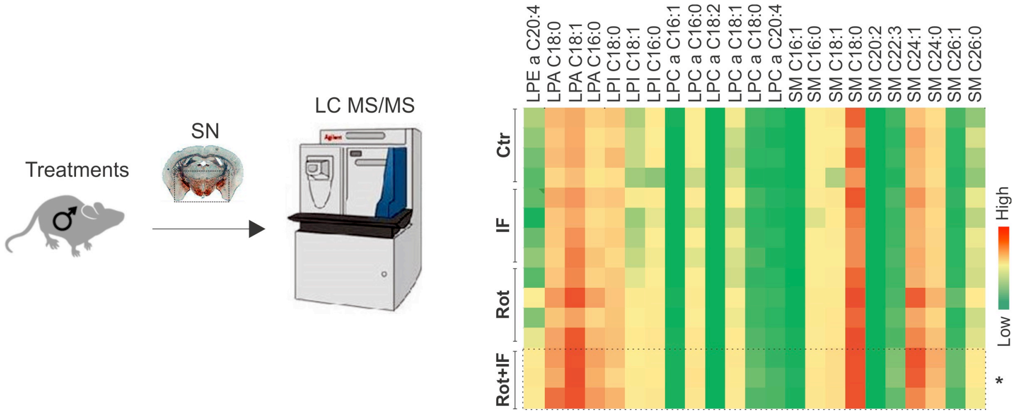

Suppl. Figure 1. Neurodegeneration is associated with altered levels of brain lipids in mice subjected to intermittent fasting.

Brain region containing *substantia nigra* (SN) were analysed by LC-MS/MS. Heat map reports the levels of detected species of lysophosphatidyl ethanolamine (LPE), lysophosphatidyl ethanolamine (LPE), lysophosphatidyl inositol (LPI), lysophosphatidic acid (LPA), lysophosphatidyl coline (LPC) and sphigomyelin (SM) for each group (asterisk (*) indicate statistically significant data as reported more in detail in Figure 4).
